# Arterial Administration of DNA Crosslinking Agents with Restraint of Homologous Recombination Repair by Intravenous Low-Dose Gemcitabine Is Effective for Locally Advanced Pancreatic Cancer

**DOI:** 10.3390/cancers14010220

**Published:** 2022-01-03

**Authors:** Hiromu Mori, Shuichi Tanoue, Ryo Takaji, Shinya Ueda, Mika Okahara, Saori Sugi Ueda

**Affiliations:** 1 Department of Radiology, Nagato Memorial Hospital, Saiki 876-0835, Japan; s-ueda@san-ai-group.org; 2 Department of Radiology, Faculty of Medicine, Oita University, Yufu 879-5593, Japan; tanoue_shuichi@med.kurume-u.ac.jp (S.T.); takajiry@oita-u.ac.jp (R.T.); okahara@oita-u.ac.jp (M.O.); 3 Department of Radiology, School of Medicine, Kurume University, Kurume 830-0011, Japan; 4 San-Ai Medical Center, Department of Radiology, Oita 870-1151, Japan; 5 Department of Radiology, Shin-Beppu Hospital, Beppu 874-8538, Japan; 6 Department of Gastroenterology, Shin-Beppu Hospital, Beppu 874-8538, Japan; irachinaosugi30285@nifty.com; 7 San-Ai Medical Center, Department of Gastroenterology, Oita 870-115, Japan

**Keywords:** locally advanced pancreatic cancer, arterial administration of DNA crosslinking agents, intravenous low-dose gemcitabine, homologous recombination repair

## Abstract

**Simple Summary:**

Pancreatic cancer is considered incurable, and most cases are detected in the advanced stages. Establishing a new, effective interventional treatment for advanced pancreatic cancer is a pressing issue. Pretreatment with gemcitabine had a restraining effect on the homologous DNA recombination repair (HRR) of DNA crosslinking, inhibiting the function of Rad51, of which expression is found to be increased in pancreatic cancer. The aim of our prospective study was to assess the potential value of the arterial administration of DNA crosslinking agents after intravenous administration of low-dose gemcitabine for patients with advanced pancreatic cancer. We confirmed, among forty-five patients with unresectable advanced pancreatic cancer, that a patient subgroup of locally advanced pancreatic cancer (LAPC, 10 patients) who underwent these treatment courses successively more than twice in the first 6 months had 33 months of overall survival, 31 months of local progression free survival, and a complete response of 40%. This treatment can be a new treatment option for LAPC.

**Abstract:**

(1) Background: Pretreatment by Rad51-inhibitory substances such as gemcitabine followed by arterial chemotherapy using antineoplastic agents causing DNA crosslink might be more beneficial for patients with locally advanced pancreatic cancers than conventional treatments. The efficacy of arterial administration of DNA crosslinking agents with pretreatment of intravenous low-dose gemcitabine for patients with unresectable locally advanced or metastatic pancreatic cancer (LAPC or MPC) is evaluated. (2) Methods: A single-arm, single-center, institutional review board-approved prospective study was conducted between 2005 and 2015. Forty-five patients (23 LAPC, 22 MPC) were included. Patients received a weekly low dose of gemcitabine intravenously for three weeks followed by arterial administration of mitomycin C and epirubicin hydrochloride at tumor-supplying arteries on the fifth or sixth week. This treatment course was repeated at 1.5-to-2-month intervals. Overall survival (OS), local progression-free survival (LPFS), and therapeutic response were evaluated. LAPC or MPC were divided according to treatment compliance, excellent or poor (1 or 2), to subgroups L1, L2, M1, and M2. (3) Results: OS of LAPC and MPC were 23 months and 13 months, respectively. The OS of LAPC with excellent treatment compliance (subgroup L1, 10 patients) was 33 months with 31 months of LPFS, and four patients (40%) had a complete response (CR). The OS of the L1 subgroup was significantly longer than those of other subgroups L2, M1, and M2, which were 17 months, 17 months, and 8 months, respectively. As Grade 3 adverse effects, severe bone marrow suppression, interstitial pneumonitis, and hemolytic uremic syndrome were observed in six (13.0%), three (6.5%), and three (6.5%) patients, respectively. (4) Conclusions: Arterial DNA crosslinking with the systemic restraint of homologous recombination repair can be a new treatment option for LAPC.

## 1. Introduction

Pancreatic cancer is considered incurable, and most cases are detected in the advanced stages [1]. The American Cancer Society reports increasing death rates for patients with pancreatic cancer, with a 5-year survival rate of only 9% [2]. The incidence rates are currently increasing in the USA, as well as in European countries, emphasizing a rising trend worldwide for this disease [3,4]. Approximately 400,000 new cases of pancreatic cancer are diagnosed annually, and death due to pancreatic ductal adenocarcinoma ranks fourth among cancer-related deaths worldwide [5]. Lack of early diagnosis and effective interventions are the major factors in the poor prognosis and dismal survival rates [6,7,8].

Gemcitabine was established as the first-line standard chemotherapy almost two decades ago and remained the standard until recently [9,10]. Recently, after 2011, OS in advanced pancreatic cancer patients has significantly improved using gemcitabine + nab-paclitaxel or FOLFIRINOX combinations when compared to the previous standard of care, gemcitabine [11,12,13,14]. Combination chemotherapy with gemcitabine or four-drug regimens followed by radiation (chemoradiotherapy) has been a feasible strategy showing relevant results in locally advanced pancreatic cancer [15,16,17,18].

To increase drug concentrations at tumor sites and to limit systemic drug exposure and its sequelae, regional arterial chemotherapy for locally advanced pancreatic cancers has been developed [19,20,21,22]. However, infusion sites of the drug have been limited to major arteries such as the celiac axis and splenic artery, and the drugs infused were mainly 5-FU. The methods vary and effectiveness is still under discussion [23].

Targeting DNA double-strand break signaling and repair has been one of the recent advances in cancer therapy. Increased expression of Rad51 in the homologous recombination repair of a DNA double-strand break (DSB) has been demonstrated in pancreatic cancer [24,25]. Wachters et al. showed that pretreatment with gemcitabine had an inhibiting effect on the homologous DNA recombination repair (HRR) of the DNA double-strand break caused by radiation or MMC, inhibiting the function of Rad51 [26].

Pretreatment by Rad51-inhibitory substances such as gemcitabine followed by arterial chemotherapy using antineoplastic agents causing DNA, DSB, or DNA crosslink might be more beneficial for patients with locally advanced pancreatic cancers than conventional treatments. In addition, to increase drug concentrations at tumor sites and to limit its sequelae such as gastrointestinal complications, selective administration of drugs into pancreatic arteries might contribute to the efficacy and safety of this treatment.

In this prospective study, the efficacy and safety of selective arterial administration of antineoplastic agents, which cause DNA crosslink, with intravenous low-dose gemcitabine used as an inhibitor of homologous recombination repair against locally advanced or metastatic pancreatic cancer are evaluated.

## 2. Materials and Methods

### 2.1. Pilot Study

Before this study, 14 patients with advanced pancreatic carcinoma (5 locally advanced carcinoma, 9 with distant metastasis) had received arterial administration of same DNA crosslinking agents: 30 mg of epirubicin hydrochloride (Farmorubicin, Kyowa Hakko Industry, Co. Ltd., Tokyo, Japan, MW 579.98) and 15 mg of mitomycin C (Mitomycin, Kyowa Hakko Industry, Ltd., Tokyo, Japan, MW 334.33) from 1998 to 2003. Average age, male/female ratio, and number of arterial administrations are 69.3 (53–80) years, 2/3, 2.0 (1–3) in patients with locally advanced carcinoma, and 63.4 (40–70) years, 4/5, 1.8 (1–4) in patients with pancreatic carcinoma with distant metastasis, respectively. 

**Figure 1:**
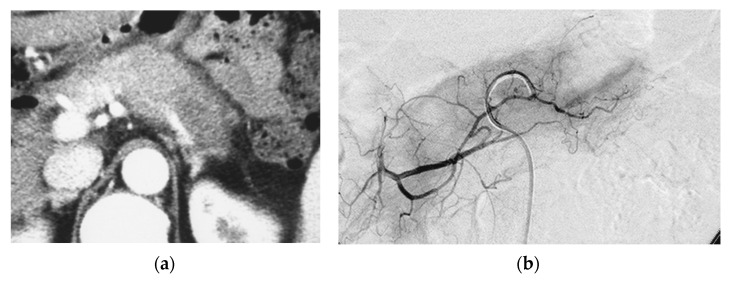

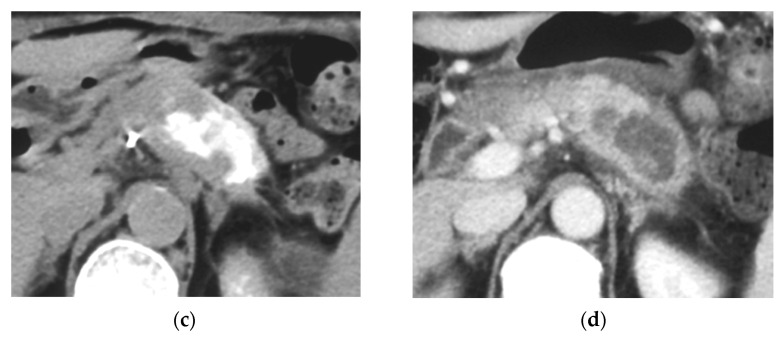
Pilot study. A 66-year-old female had locally advanced pancreatic carcinoma at body and tail. An arterial administration of DNA crosslinking agents achieved good tumor necrosis. Patient had died after 12 months. Dense retention (dense contrast materials in area >50% of tumor). (**a**) Postcontrast CT shows a mass at the pancreatic tail. A hypodense mass occupies pancreas body and tail encasing splenic vein (arrow). (**b**) Superselective dorsal pancreatic angiography shows severe encasement of the transverse pancreatic artery. Arrows indicate the presence of a hypovascular mass. (**c**) CT immediately taken after administration of the drugs mixed with contrast media shows dense contrast retention in area >50% of tumor. (**d**) Follow-up CT two weeks after the superselective infusion shows massive necrosis in the tumor.

Although good tumor necrosis was achieved in every patient where anticancer drugs mixed with contrast media were accumulated on post-procedure CT, median survival time of patients with pancreatic carcinoma and patients with pancreatic carcinoma with distant metastasis were 7.0 ± 2.50 months and 5.0 ± 0.94 months, respectively (Figure 1). No severe adverse effects were observed.

### 2.2. Study Design

This prospective, single-center, single-arm study was planned after publication of Wachters et al. [26], which stated that pretreatment by Rad51-inhibitory substances, namely gemcitabine, followed by DNA double-strand break or DNA crosslinking was effective for cancer cells. The protocol was approved by the local ethics committee, and all patients gave informed consent. All procedures performed in studies involving human participants were in accordance with the ethical standards of the institutional and/or national research committee and with the 1964 Helsinki declaration and its later amendments or comparable ethical standards. Patients were enrolled from January 2005 to December 2015 for selective administration of antineoplastic agents to arteries, which supply pancreatic cancer with intravenous systemic low-dose gemcitabine. 

### 2.3. Patients

Patients were eligible for the study if they were 18 years of age or older and had received a diagnosis of advanced unresectable pancreatic ductal adenocarcinoma. The diagnosis was made based on biopsy, cytology of fine needle biopsy, cytology of pancreatic juice, multidetector computed tomography (MDCT) findings, and elevation of tumor markers including, primarily, CA19-9. Performance status was 0, 1, or 2 with an estimated life expectancy of more than 3 months. The main exclusion criteria were clinical ascites, severe alteration of liver function, uncontrolled severe coagulation disorders, and previous systemic chemotherapy or radiation therapy and/or pancreatic partial resection for pancreatic cancer. 

### 2.4. Procedure and Treatment

#### 2.4.1. Treatment Course

The treatment course, consisting of 3 intravenous administrations of weekly gemcitabine (three weeks) and an arterial administration of epirubicin hydrochloride and mitomycin C at 5th or 6th week, was repeated at 1.5-to-2-month intervals after bone marrow suppression recovered. It was repeated until pancreatic mass disappeared on imaging, until severe adverse effects such as severe bone marrow suppression were observed, or until patient expressed his/her preference to discontinue the treatment course for any reason. The maintenance systemic chemotherapy then started after the treatment course discontinued.

#### 2.4.2. Systemic Chemotherapy by Intravenous Low-Dose Gemcitabine

Before the pancreatic arterial chemotherapy, patients were administered an intravenous infusion of gemcitabine at a dosage of 150–500 mg/m^2^ weekly on day 1, 8, and day 15 (1st, 2nd, and 3rd week). Dosage started with 500 mg/m^2^ and decreased depending on patients’ degree of bone marrow suppression after one treatment. 

#### 2.4.3. Selective Arterial Administration of Agents Which Cause DNA Double-Strand Break or DNA Crosslink into Pancreatic Arteries and Adrenal Arteries

Arterial administration of chemotherapeutic agents that cause DNA double-strand break or DNA crosslink was scheduled around day 29 or 36 (5th week or 6th week) followed by 2–4 weeks for observation.

##### Identifying Supplying Arteries to Pancreatic Cancer

A catheter was inserted by femoral artery puncture, and celiac and superior mesenteric arteriography were first conducted to examine arterial and portal invasion of pancreatic cancer. Selective catheterization in pancreatic arteries was performed to confirm the arterial supply to pancreatic cancer under CT guidance based on the arterial anatomy and territories [27]. A 4-Fr parental catheter with a 2-Fr microcatheter was used in catheterization. For extra-pancreatic extension of cancer, we identified supplying arteries using multidetector CT as guidance among retroperitoneal arteries such as adrenal arteries.

##### Method of Arterial Administration of Anticancer Agents

Anticancer agents that cause DNA double-strand break or DNA crosslink used were 30 mg of epirubicin hydrochloride (Farmorubicin, Kyowa Hakko Industry, Co. Ltd., Tokyo, Japan, MW 579.98) and 15 mg of mitomycin C (Mitomycin, Kyowa Hakko Industry, Ltd., Tokyo, Japan, MW 334.33). They were diluted in 7.5 mL of physiological saline and mixed with 7.5 mL of contrast agent (Iopamilon 370, Bayer Co. Ltd., Osaka, MW 777.09). Depending on body weight of patients, 10 mL to 15mL of this solution was administered from supplying arteries. Doses per artery was divided according to proportions of opacification within mass on CT during selective arteriography. Doses of anticancer agents were 0.17–0.25 mg/Kg of mitomycin C and 0.33–0.5 mg/kg of epirubicin hydrochloride.

The manual pulse method was used for injection, taking approximately 5–10 min. When a number of small arterial branches, which could not be catheterized, were involved as tumor vessels, a 4F sheath was changed to 5F guiding sheath, having 0.080 inner diameter (Axcelguid 5F, Medikit Co. ltd., Tokyo, Japan). After parental catheter was advanced into the proximal parent artery, the periphery of parental artery was occluded by micro-balloon catheter (2.7–3F) and administration of anticancer agents was performed using parallel microcatheter at origin of feeding artery.

Metastatic tumors were not targeted for arterial administration of anticancer agents in any treatment session in all patients because the entire amount of anticancer agent was administered to only pancreatic tumors to avoid adverse effects, especially of bone marrow suppression.

#### 2.4.4. Maintenance of Systemic Chemotherapy

After the treatment course discontinued, systemic chemotherapy using low-dose gemcitabine was carried out. In cases where the patient showed severe adverse effects, such as severe bone marrow depression or interstitial pneumonitis caused by systemic chemotherapy by gemcitabine, TS-1 capsule (tegafur, gimeracil, and oteracil potassium combination capsule drug, Taiho Pharmaceutical Co., Ltd., Tokyo, Japan) was used as the second-line systemic chemotherapy. Systemic chemotherapy was carried out chiefly on an outpatient basis.

### 2.5. Assessments

At baseline, all patients underwent a complete medical history and physical examination, assessment of performance status, routine laboratory studies, CEA/CA19-9 levels, and vital signs. Tumor assessments included postcontrast CT, magnetic resonance imaging (MRI).

#### 2.5.1. Judging Completeness or Incompleteness of Arterial Administration of Chemotherapeutic Agents

Two interventional radiologists judged if arterial administration of chemotherapeutic agents was performed from all supplied arteries (complete) or not (incomplete), evaluating images of axial, coronal, and sagittal reconstructed CT taken during arterial administration of contrast medium at 10 s and 30 s.

#### 2.5.2. Survival Times

For progression-free survival (PFS), progressive disease was defined as either pancreatic tumor progression or the progression of distant metastatic or lymph node disease. Except for the arterial treatment, patients were basically seen in outpatient clinic. Follow-up visits consisted of clinical assessment and CT to assess tumor progression. Once local progressive disease was documented, patients underwent follow-up for survival only.

#### 2.5.3. Tumor Response

Tumor response was determined according to the response evaluation criteria in solid tumors (RECIST) 1.1 criteria [28] by means of postcontrast CT at 6 months after the first treatment course was completed. Tumor response was defined as complete response (CR) or a partial response (PR). Tumor control was defined as a complete response (CR), a partial response (PR), or stable disease (SD). CR was defined as no tumor evidence on contrast enhanced CT, combined with normalized CA19-9 and other tumor markers such as Span-1 and DUPAN-2.

#### 2.5.4. Safety Assessment

Safety assessment was performed before each cycle with the use of the National Cancer Institute Common Terminology Criteria for Adverse Events (CTCAE v4.0) [29]. Toxicities were graded according to Common Terminology Criteria for Adverse Events (CTCAE) 4.0. Vital signs, laboratory studies, ECOG, and toxicities were assessed prior to each treatment application.

#### 2.5.5. Statistical Analysis

The primary endpoints were to evaluate overall survival (OS) and the progression-free survival (PFS). The secondary endpoints were to assess therapeutic response, adverse events, and clinical benefit response. Overall survival times were estimated by the Kaplan–Meier survival analysis. Cause-specific OS was also estimated because one patient died of acute myocardial infarction 36 months after turning CR.

All analyses were performed on an intention-to-treat basis. LPFS was measured from the date of diagnosis as pancreatic cancer to local disease progression, and OS was measured from the date of diagnosis as pancreatic cancer by imaging to death, by the Kaplan–Meier method. Survival probabilities were compared using the log-rank test. All analyses were performed on an intention-to-treat basis.

Univariate statistical analysis was conducted for tumor response and toxic effects as well as patients’ characteristics, including age, gender, macroscopic growth pattern, disease extent, and elevated CEA and CA19-9 levels. Lifetime statistical analysis and the Fisher’s exact test were used to compare the hazards and response rates for the patients. A two-sided *p* value < 0.05 was considered to prove significance for all tests. Data analysis was performed using the Statistical Package for the Social Sciences (SPSS, version 17.0) and Statistical Analysis System (SAS, version 9.4).

## 3. Results

### 3.1. Patient’s Characteristics

From January 2005 to December 2015, 45 patients (25 men, 20 women) with advanced pancreatic cancer showing local invasion or metastasis were enrolled. The median age was 68.46 ± 11.20 years (range 36–89 years of age) at the start of treatment. The locally advanced cancer group consisted of 23 patients with arterial or portal venous invasion, and the metastatic advanced cancer group consisted of 22 patients with hepatic metastasis or distant nodal metastasis and peritoneal dissemination. The median follow-up period was 23.3 ± 24.1 (6–144) months (Table 1).

### 3.2. Treatment Compliance

All patients underwent a treatment course (3 weeks of venous gemcitabine followed by arterial administration of mitomycin C and epirubicin hydrochloride) more than once in the first 6 months after being diagnosed. Thirty-eight patients received at least two treatment courses. Eight patients received one treatment course. One hundred and eighty-two courses were applied in total (median 3.96 per patient; range 1–9).

The treatment course was repeated at intervals of 1.5 to 2 months until the pancreatic mass disappeared on imaging along with the normalization of the level of tumor markers (n = 5), until severe adverse effects such as severe bone marrow suppression were observed (n = 31), or until other reasons to discontinue appeared (*n* = 10).

### 3.3. Patient Subgrouping According to Treatment Compliance

We categorized the locally advanced cancer group as group L (*n* = 23) and patients with distant metastasis as group M (*n* = 22). We then subclassified them into groups 1 and 2 according to technical successfulness of arterial administration of chemotherapeutic agents and treatment compliance of patients as follows, because treatment compliance in the first 6 months (after being diagnosed as having pancreatic cancer) seemed crucial for prognosis. 

Group 1 was populated by patients who fulfilled all of three conditions, including: (1) treatment courses were successively performed more than twice in the first 6 months, (2) arterial administrations of chemotherapeutic agents seemed completely performed at all of the supplying arteries to the tumor, and (3) systemic chemotherapy was not interrupted for more than 6 months after the last treatment course (excellent treatment compliance group).

Group 2 was populated by patients who did not fulfill the three conditions described above. Namely, treatment courses were performed once in the first 6 months or were not successively performed in the first 6 months, having an interruption of more than 2 months, arterial administrations of chemotherapeutic agents failed to be performed at all the supplying arteries to the tumor, and systemic chemotherapy was discontinued during 6 months after the last treatment course (poor treatment compliance group).

We had four subgroups consisting of L1, L2, M1, and M2. In the localized advanced cancer group (L), L1 consisted of 10 patients, and L2 consisted of 13 patients. On the other hand, in the metastatic advanced cancer group (M), M1 consisted of 10 patients, and the group of discontinued systemic chemotherapy (M2) consisted of 12 patients.

The average number of treatment courses (3 weeks of intravenous gemcitabine followed by arterial administration of mitomycin C and epirubicin hydrochloride) was 4.9 (range 2–9) in L1 and 2.8 (range 1–8) in L2 in the localized advanced cancer group. For the metastatic advanced cancer group, it was 3.6 (range 2–9) in M1 and 1.7 (range 1–4) in M2.

### 3.4. Survival and Disease Progression

The final analysis was performed in September 2018, which was 14 years after the first patient had been enrolled and 3 years after the last patient had been enrolled. At that point, two patients had been alive (12 years and 8 years after diagnosis, respectively), and 44 patients had died. Forty-three patients died of progression of pancreatic cancer or metastatic pancreatic cancer, and one patient died of acute myocardial infarction at 3 years after reaching the complete response (CR) of pancreatic cancer.

**Figure 2:**
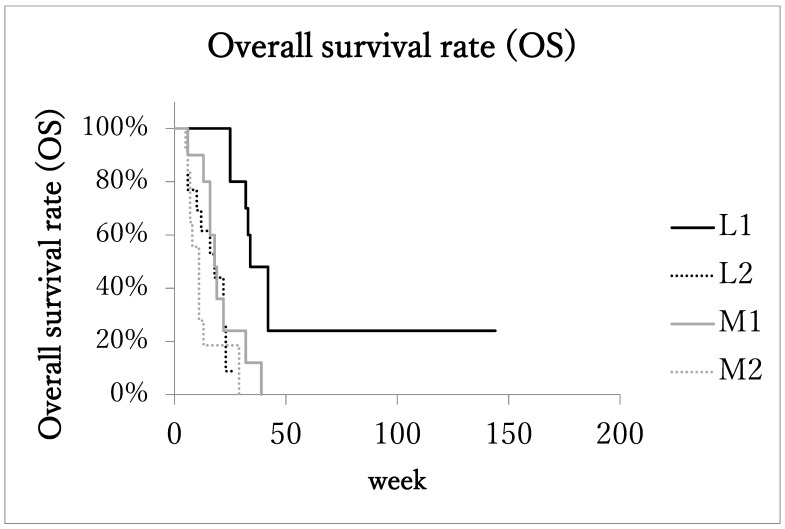
Kaplan–Meier estimates of overall survival (OS). Patients with localized advanced cancer with excellent treatment compliance (L1) had higher survival rates than those of the other 3 subgroups.

Median overall survival time (OS) of patients with the localized advanced cancer (L1 + L2) was 23 months (range: 5–144 months, IQR: 13.75–32.75, 95%CI: 15.4–40.3). The OS of patients with the localized advanced cancer with excellent treatment compliance (L1, 10 patients) was 33 months (range: 25–144 months, IQR: 32–42, 95%CI: 16.8–74.3; Figure 2), and that of patients with the localized advanced cancer with poor treatment compliance (L2, 13 patients) was 17 months (range: 5–27 months, IQR: 9–22.3, 95%CI: 11.0–21.8).

On the other hand, in the metastatic advanced cancer group (M1 + M2), the median overall survival time (OS) was 13 months (range: 5–39 months, IQR: 7–17, 95%CI: 10.2–17.8). In the metastatic advanced cancer group with excellent treatment compliance (M1, 10 patients), OS was 17 months (range: 6–39 months, IQR: 16–19, 95%CI: 11.6–25.2), and in the group with poor treatment compliance (M2, 12 patients) it was 8 months (range: 5–29 months, IQR: 6.5–11, 95%CI: 5.9–15.0). The OS of subgroup L1was significantly longer than the other three subgroups.

**Figure 3:**
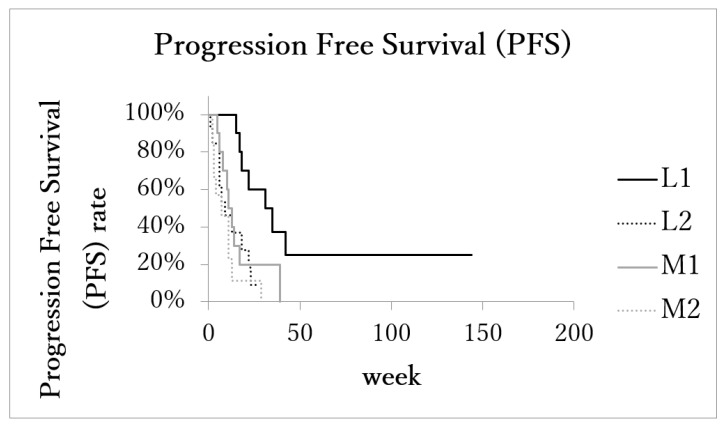
Kaplan–Meier estimates of local progression-free survival (LPFS). Patients with localized advanced cancer with excellent treatment compliance (L1) had higher survival rates than those of the other 3 subgroups.

The median cause-specific survival time of patients with the localized advanced cancer (L1 + L2) was 23 months (range: 5–144, IQR: 13–32, 95%CI: 14.1–40.2). In the metastatic advanced cancer group (M1 + M2), LPFS was 13 months (range: 5–39, IQR: 7–17, 95%CI: 10.2–17.8). The median cause-specific survival times of subgroups L1, L2, M1, and M2 were 33 months (range: 25–144, IQR: 30.2–36, 95%CI: 12.6–79.4), 17 months (range: 5–27, IQR: 9–22.3, 95%CI: 11.0–21.8), 17 months (range: 6–39, IQR: 16–19, 95%CI: 11.6–25.2), and 8 months (range: 5–29, IQR: 6.5–11, 95%CI: 5.9–15.0), respectively. The median cause-specific survival time of subgroups L1 was significantly longer than the other three subgroups (Figure 3).

**Figure 4:**
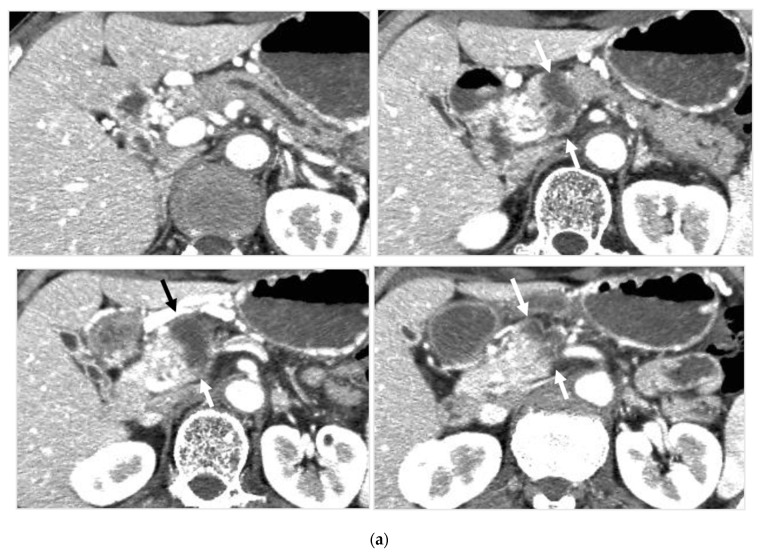

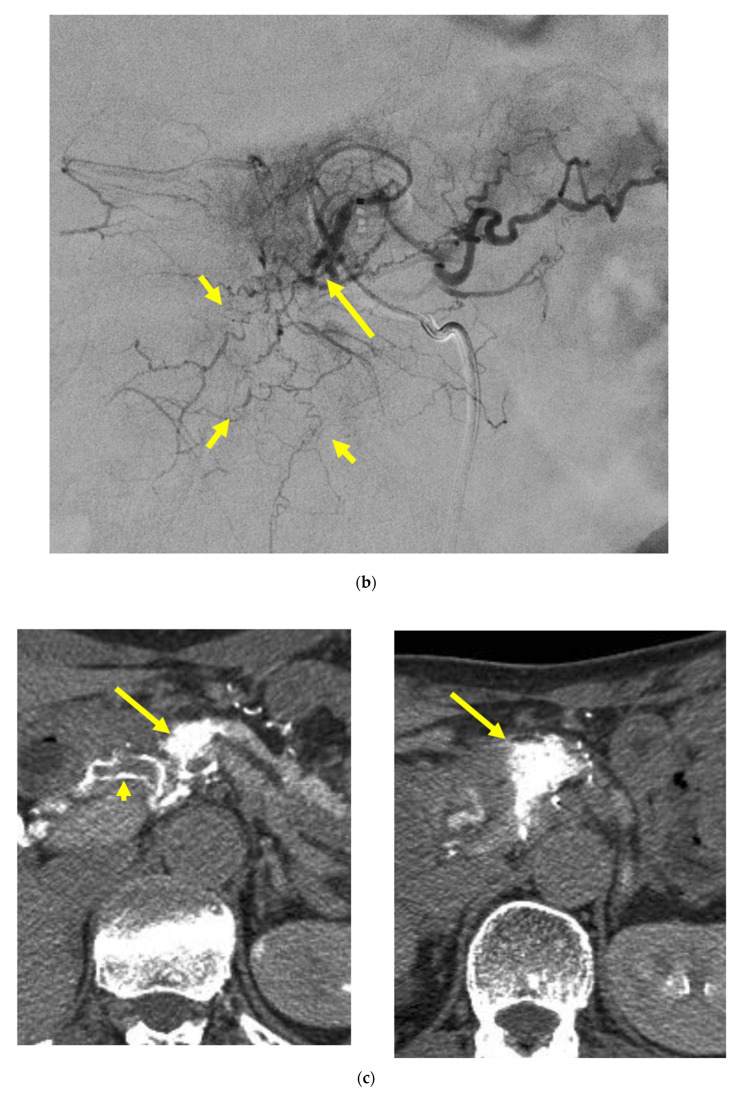

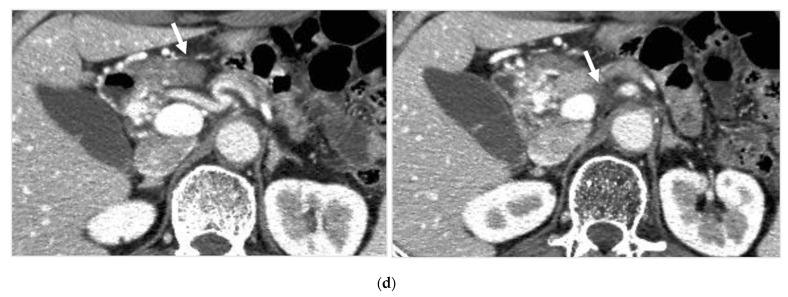
Locally advanced cancer of the pancreas with excellent treatment compliance. A 78-year-old man with locally advanced pancreatic carcinoma at pancreatic body received 4 treatment courses in the first year, resulting in CR and continued life for 144 months after diagnosis. (**a**) Postcontrast CT (arterial phase) immediately before the first treatment course. A mass of low attenuation with maximal diameter of 3.5 cm (arrows) was observed at the proximal portion of the pancreatic body occluding splenic vein and extending to the common hepatic artery, splenic artery, and superior mesenteric artery. (**b**) The first arterial administration of chemotherapeutic agents (mitomycin C and epirubicin hydrochloride) mixed with contrast media after 3 weekly intravenous low-dose gemcitabine. Selective dorsal pancreatic angiography shows occlusion of the distal dorsal pancreatic artery (arrow) and severe encasement of its branches depicting a hypovascular mass (short arrows). (**c**) CT taken during dorsal pancreatic angiography show a dense opacification of most parts of the tumor (arrows). Note opacification of wall of common hepatic artery (short arrow). Twelve milliliters of contrast media (20 mL) mixed with chemotherapeutic agents were administrated at the dorsal pancreatic artery. Two milliliters of a mixture of contrast media and chemotherapeutic agents were also administrated at the pancreatica magna artery (not shown). (**d**) Postcontrast CT (arterial phase) after two treatment courses showed a marked decrease in the size of the tumor (arrow), which had no enhancement effect until the parenchymal phase. Note the atrophy of the pancreatic body and the tail and splenic vein. After this arterial administration, all elevated tumor markers became normal, including CA19-9 (142.7 U/mL → 7.9 U/mL), elastase-1 (1340 ng/dl → 121 ng/dL), and SPAN-1 (110 U/mL → 5.7 U/mL). The patient showed no sign of recurrence for 11 years.

The median local progression-free survival (LPFS) of patients with the localized advanced cancer (L1 + L2) was 17.5 months (range: 1–144 months, IQR: 7.5–26, 95%CI: 10.1–36.0). In the metastatic advanced cancer group (M1 + M2), LPFS was 8 months (range: 2–39 months, IQR: 4–13, 95%CI: 6.2–14.5). LPFS of subgroups L1 (*n* = 11), L2 (*n* = 11), M1 (*n* = 10), and M2 (*n* = 10) was 31 months (range: 15–144, IQR: 18–35, 95%CI: 8.8–70.6), 9.5 months (range: 1–27, IQR: 6–19, 95%CI: 6.5–17.5), 11 months (range: 5–39, IQR: 8–14, 95%CI: 5.8–21.5), and 4 months (range: 2–29, IQR: 3–9, 95%CI: 2.1–12.7), respectively. LPFS of subgroup L1 was significantly longer than the other three subgroups (Figure 4).

### 3.5. Tumor Response

Tumor control was achieved in 37 of 46 patients (80.4%), including five complete responses (CR) and 32 partial responses (PR). Stable disease (SD) was not observed, and progressive disease (PD) occurred in nine patients (19.6%). In the subgroups, L1 had 4CR and 7PR, L2 had 12PR and 1PD, M1 had 1CR, 9PR, and 1PD, and M2had had 6PR and 6PD.

The overall response rate of patients grouped in L1, L2, and M1 was higher than that of patients in M2. Among patients of CR, two were alive (98 months, 144 months after diagnosis with pancreatic cancer) having active lives, one died of acute myocardial infarction without regrowth of the pancreatic mass in 42 months, and one died of duodenal hemorrhage with tumor regrowth at 42 months after 35 months of the local-progression-free period. In one patient with omental metastasis, whose pancreatic tumor was CR, metastasis progressed to the peritoneal cavity and lungs, and the patient died at 39 months after diagnosis. 

Metastatic tumors that were not targeted for arterial administration of anticancer agents in any treatment session in all patients had gradually enlarged during the observation course.

Surgical evaluation of pancreatic tumors after arterial administration of anticancer agents was not performed in patients with locally advanced disease.

### 3.6. Treatment Toxicity

Mild nausea and general fatigue were the most common adverse events that occurred in 32 (69.6%) and 36 (78.3%) patients, respectively. Severe bone marrow suppression including anemia, leucopenia, and thrombopenia was observed in six patients (13.0%). Interstitial pneumonitis, hemolytic uremic syndrome, and severe vomiting were observed in three patients (6.5%), three patients (6.5%), and one patient (2.2%), respectively.

There was no association of pancreatitis nor inflammation of the duodenum and jejunum.

## 4. Discussion

Pancreatic cancer has been considered incurable, and most cases are detected in the advanced stages [1]. Recently, overall survival in advanced pancreatic cancer patients is significantly improved using the gemcitabine + nab-paclitaxel or FOLFIRINOX combinations, or chemoradiotherapy, showing excellent results of OS up to 26 months for locally advanced pancreatic cancer patients, and they were thought to be feasible strategies for locally advanced pancreatic cancer [14,15,16,17,18]. However, progression-free time is still not long enough and new breakthroughs are needed for patients with locally advanced pancreatic cancer.

Although pancreatic cancer is generally understood as a hypovascular tumor either on an angiogram or on a postcontrast CT, CT taken during selective injection of contrast material into the pancreatic artery such as the dorsal pancreatic artery showed substantial opacification and retention of the contrast material within the pancreatic cancer, as well as in other cancers [30]. It also was confirmed that there were molecular interactions between anticancer agents and iodinated contrast media [31]. Based on these findings, we decided to attempt the selective injection of anticancer agents mixed with iodinated contrast material into the pancreatic artery, which should allow substantial retention of anticancer agents within the pancreatic cancer, achieving a better anticancer effect. In our method of arterial administration of antineoplastic agents, the phenomenon that the leakiness of the tumor vasculature makes nano-sized particles that spontaneously accumulate within tumor, which is known as the enhanced permeation and retention (EPR) effect [32,33], might contribute to the retention of the contrast material/antineoplastic agents complex within the tumor in our method.

Arterial chemotherapy, which increases drug concentrations at tumor sites and limits systemic drug exposure and its sequelae, should be one method; however, previously reported methods had limited infusion sites of major arteries, and the drugs infused were mainly 5-FU [19,20,21,22,23]. Our strategy was pretreatment by Rad51-inhibitory substances by intravenous low-dose gemcitabine followed by arterial administration of antineoplastic agents causing DNA, DSB, or DNA crosslink, which might be more beneficial for patients with locally advanced pancreatic cancers than other treatments previously reported. Selective administration of antineoplastic agents causing DNA crosslink at all supplying arteries to the pancreatic tumor, especially administration to arteries such as adrenal arteries, to cover a tumor of extra-pancreatic extension was important to achieve effectiveness. This strategy of treatment may be applicable to so-called hypovascular cancers like pancreatic cancer. Of course, the effect might be depending on the Enhanced Permeation and retention (EPR) effect of individual tumor.

Selective arterial administration of antineoplastic agents needs meticulousness for interventional radiologists because these supplying arteries are of small caliber with many anatomical variations in most cases. CT during arterial injection of contrast material made it easier to cover all parts of pancreatic cancers. No adverse effect was not observed in the stomach, duodenum, and jejunum, even if pancreatic tumor locations adjacent to these G-I tracts show the safety of this treatment. This feasibility to apply to pancreatic cancer extending or neighboring the duodenum or jejunum is an advantage compared to radiation therapy and surgical resection.

In this study, the median overall survival time (OS) of patients with the localized advanced cancer with excellent treatment compliance (L1, 10 patients) was 33 months with four CR patients, and that of patients with the localized advanced cancer with poor treatment compliance group (L2, 13 patients) was 17 months. On the other hand, in the metastatic advanced cancer group with excellent treatment compliance (M1, 10 patients), the median overall survival time was 17 months, and in the group of poor treatment compliance (M2, 12 patients) it was 8 months. The median OS was significantly longer than the other three subgroups. This result indicates that this treatment, arterial administration of DNA crosslinking agents with inhibition of homologous recombination repair by intravenous low-dose gemcitabine, is effective for locally advanced pancreatic cancer, and we can expect CR, but effectiveness is not enough for metastatic advanced pancreatic cancer. These promising survival results can be further improved with modern multi-agent chemotherapy regimens such as FOLFIRINOX in the future.

Compared to the pilot study, our treatment seems better than treatment by arterial administration only; however, this difference of survival time may be bridged by systemic chemotherapy using the gemcitabine + nab-paclitaxel or FOLFIRINOX combinations. To have an excellent treatment compliance, careful monitoring of bone marrow function and timely use of the granulocyte colony stimulating factor (GCSF) as well as a blood transfusion might be essential.

Administering DNA crossing agents at all supplying arteries to the tumor is essential to this treatment and seems not so easy for interventional radiologists now. This is one limitation of this treatment, as well as CT-guided angiography being needed, which is an indication that this treatment might be limited to patients with the localized advanced cancer, excluding patients with metastatic advanced pancreatic cancer.

Targeting DNA double-strand break signaling and repair has been one of the recent advances in cancer therapy [34]. Increased expression of Rad51 in the homologous recombination repair of a DNA double-strand break has been demonstrated in pancreatic cancer [24,25]. Wachters et al. showed that pretreatment with gemcitabine had an inhibiting effect on homologous DNA recombination repair (HRR) of a DNA double-strand break (DSB) caused by radiation or MMC, inhibiting function of Rad51 [26]. It is well known that gemcitabine works as antimetabolites to interfere the specific phase of cell cycle. Most probable explanation of this RAD51 inhibitory function of gemcitabine is that function of RAD51, which is formed in the S or G2 phases and regulates cell cycle progression by preserving the G2/M transition, is inhibited because gemcitabine inhibits DNA synthesis, i.e., stoppage of cell cycle progression.

In addition to gemcitabine, there is a growing list of Rad51-inhibitory substances and therapeutics to inhibit HRR. These include nucleoside and base analogs such as gemcitabine, TAS-106 [35], and gimeracil [36], as well as other antimetabolites such pentoxifylline and caffeine [37], and the tyrosine kinase inhibitors imatinib and erlotinib [38,39,40]. It was confirmed when enhanced cell killing effects by gemcitabine in radiotherapy were observed for pre-treatment but not for post-treatment with gemcitabine [41]. Clinically, we used gemcitabine in this study because it was the only drug that was covered by insurance during the study period in our country.

In regards to low-dose gemcitabine, neoadjuvant chemoradiotherapy with a low dose of gemcitabine of 50–150 mg/m^2^ per week has proved its effectiveness for locally advanced pancreatic cancer [42,43,44,45]. Various low doses of gemcitabine were administered for gemcitabine-based radiotherapy, and despite this low dose, excellent results were achieved. Treatment of intravenous low-dose gemcitabine has a merit for controlling cancer that extends outside the pancreas, including lymph nodes as well as enhancing the effect of the DNA crosslink produced by arterial administration of MMC and epirubicin.

In the future, due to progress in genetic expression analysis by microarray techniques, tailored treatment that corresponds to the individual genetic characteristics of the patient by understanding the sensitivity, tolerance mechanism, and adverse effects of anticancer drugs might emerge. Recent reports have mentioned that chip-based digital PCR will make it possible to predict patients’ response to chemotherapeutic agents by analyzing proteins such as hENT1, dCK, ERCC1, SPARC mRNA expression, and BRCA2 mutation [46,47,48,49]. At that time, the role of arterial administration of antineoplastic drugs to pancreatic cancer will become different. Arterial administration of cisplatin might be effective for the patients with low ERCC1 expression. Arterial administration of DNA crosslinking agents such as cisplatin or mitomycin C will be significantly effective for patients with inactivated BRCA2. It might be one possible regime for advanced pancreatic cancers for which downstaging is first achieved by systemic chemotherapy using agents with a predicted good response, followed by arterial administration of the same agents in expectation of CR.

The limitations of our study are as follows: 1. it was a single-center, single-arm study; 2. there was no control study of intravenous low-dose gemcitabine; 3. there was a small number of patients; 4. there was no randomized trial. Among the patients with advanced pancreatic cancer who were indicated to receive chemotherapy such as the gemcitabine + nab-paclitaxel or FOLFIRINOX combinations, we enrolled patients who gave informed consent to receive our treatment of arterial administration of DNA crosslinking agents with intravenous low-dose gemcitabine.

## 5. Conclusions

Pretreatment by Rad51-inhibitory substances, namely intravenous low-dose gemcitabine, followed by arterial administration of DNA crosslinking agents into pancreatic arteries was beneficial for patients with locally advanced pancreatic cancers, based on the satisfactory survival time and high rate of CR patients.

## Figures and Tables

**Table 1:** Baseline characteristics of patients, number of treatment courses, time of uninterrupted systemic chemotherapy, and tumor response.

Groups of PCA	Locally Advanced PCA		Metastatic Advanced PCA	
Subgroups of PCA	L1	L2	M1	M2
Characteristics	n = 10	n = 13	n = 10	n = 12
Age (years)	66.4 (43–82)	75.0 (42–89)	63.5 (36–87)	67.5 (56–76)
Gender (Male/Female)	8:2	5:8	6:4	7:5
Location of mass (head:body:tail)	7:3:0	9:3:1	3:7:0	5:3:4
Maximum tumor size in average cm	3.6 (3.0–4.2)	4.2 (3.0–5.5)	4.7 (3.5–6.0)	4.9 (4.0–5.8)
Performance status 0	5	2	2	2
Performance status 1	4	7	4	4
Performance status 2	1	4	4	4
Number of treatment courses	4.9 (2–9)	2.8 (1–8)	3.6 (2–9)	1.7 (1–4)
Time of uninterrupted systemic chemotherapy after the last treatment course (months)	11 (5–24)	5.2 (1–12)	8.3 (4–17)	7.5 (2–28)
Tumor response (RECIST) CR/PR/SD/PD	4/6/0	0/12/1	0/9/1	0/6/6

Note: PCA = pancreatic cancer; L1, M1 = patients who fulfilled all of three conditions, including: (1) treatment courses were successively performed more than twice in the first 6 months, (2) arterial administrations of chemotherapeutic agents seemed completely performed at all of the supplying arteries to the tumor, and (3) systemic chemotherapy was not interrupted for more than 6 months after the last treatment course (excellent treatment compliance group); L2, M2 = patients who did not fulfill the 3 conditions (poor treatment compliance group).
